# Natural Occurrence of *Alternaria* Toxins in the 2015 Wheat from Anhui Province, China

**DOI:** 10.3390/toxins8110308

**Published:** 2016-10-26

**Authors:** Wenjing Xu, Xiaomin Han, Fengqin Li, Lishi Zhang

**Affiliations:** 1 Key Laboratory of Food Safety Risk Assessment, Ministry of Health, China National Center for Food Safety Risk Assessment, Beijing 100021, China; wenjingxu_vip@163.com (W.X.); hanxiaomin@cfsa.net.cn (X.H.); 2 Institute of Nutrition and Food Hygiene, West China College of Public Health, Sichuan University, Chengdu 610041, China

**Keywords:** *Alternaria* toxins, wheat, China, HPLC-MS/MS

## Abstract

The exposure to *Alternaria* toxins from grain and grain-based products has been reported to be related to human esophageal cancer in China. In this study, a total of 370 freshly harvested wheat kernel samples collected from Anhui province of China in 2015 were analyzed for the four *Alternaria* toxins tenuazonic acid (TeA), tentoxin (TEN), alternariol (AOH) and alternariol monomethyl ether (AME) by high performance liquid chromatography-tandem mass spectrometry method (HPLC-MS/MS). TeA was the predominant toxin detected followed by TEN, AOH and AME. The concentrations of the four *Alternaria* toxins varied geographically. The samples from Fuyang district showed higher TEN concentration levels than the other regions studied (*p* < 0.05). Furthermore, 95% (352/370) of the wheat samples were positive for more than one type of *Alternaria* toxins. Positive correlation was observed between concentration levels of TeA and TEN, AOH and AME, TeA and AOH, and the total dibenzopyrone derivatives (AOH + AME) and TeA. Results indicate that there is a need to set the tolerance limit for *Alternaria* toxins in China, and more data on the contamination of these toxins in agro-products is required.

## 1. Introduction

*Alternaria* species are pathogenic, endophytic and saprophytic fungi that have been reported to cause extensive spoilage of crops such as grain, tomato, potato, citrus, apple and sunflower seed in the field or after harvest [1,2,3,4,5,6,7,8]. The invasion of agricultural commodities by *Alternaria* species are universal and result not only in considerable loss of crops due to decay but also contamination by *Alternaria* toxins. *Alternaria* species can produce more than 70 toxins, of which only a small proportion have been chemically characterized. These *Alternaria* toxins can be classified into three main categories: the dibenzo-α-pyrones, which include alternariol (AOH), alternariol monomethyl ether (AME), altenuisol (AS) as well as altenuene (ALT); tetramic acid derivatives including tenuazonic acid (TeA); and the perylene derivatives altertoxins I, II and III (ATX I, II and III) [9,10]. *Alternaria* toxins show cytotoxic activity among mammalian cells, and fetotoxicity and teratogenicity among mice and hamsters [11]. Some individual mycotoxins such as AOH and AME, though not acutely toxic, are mutagenic and genotoxic in various in vitro systems [12]. TeA is considered to be of the highest toxicity among the *Alternaria* toxins, and has been proven to be toxic to several animal species, e.g., mice, chicken and dogs [8].

The key contribution of dietary exposure to *Alternaria* toxins is from grain and grain-based products, especially wheat products [12]. Wheat is one of the most important foods for the Chinese population. More than half of Chinese people live on wheat as a stable food, particularly in the north part of China [13]. For example, Anhui province is a major wheat-producing region and a major outbreak of human intoxication attributed to the consumption of wheat-based foods contaminated with *Fusarium* toxins (mainly deoxynivalenol) occurred in 1991, with over 130,000 people being affected by gastrointestinal disorder [14]. Our recent research showed that *Alternaria* species were the predominant invading fungi in wheat collected from Anhui province in 2015 [15]. Further, the intake of grains contaminated by *Alternaria* toxins has been related to human esophageal cancer in some areas of China [16]. Therefore, the *Alternaria* toxins in wheat represent a potential hazard to the Chinese population.

Due to limited data available on natural occurrence of these toxins in foods and their toxicity to humans and animals, regulations for *Alternaria* toxins in food and feed have not been set nationally or internationally. The purpose of this study is to elucidate the natural occurrence of four major *Alternaria* toxins, namely TeA, tentoxin (TEN), AOH and AME, using the wheat samples from Anhui province collected in 2015. The results obtained will provide a scientific basis for assessing the impact of *Alternaria* toxins on Chinese public health resulting from consumption of wheat products.

## 2. Results

### 2.1. High Performance Liquid Chromatography-Tandem Mass Spectrometry (HPLC-MS/MS) Method Validation

**Figure 1:**
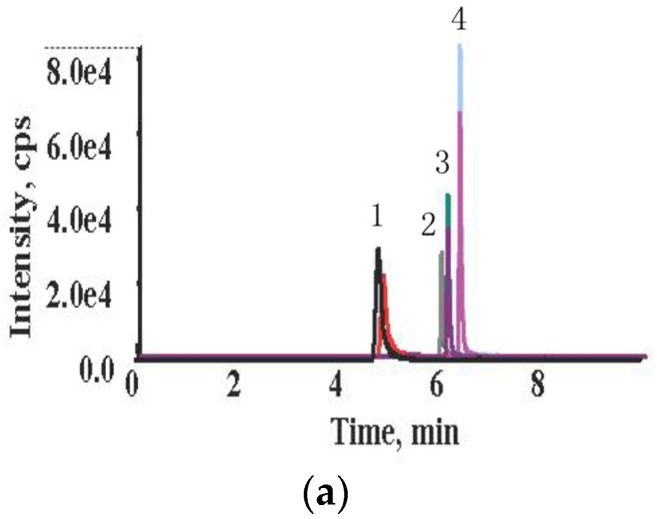

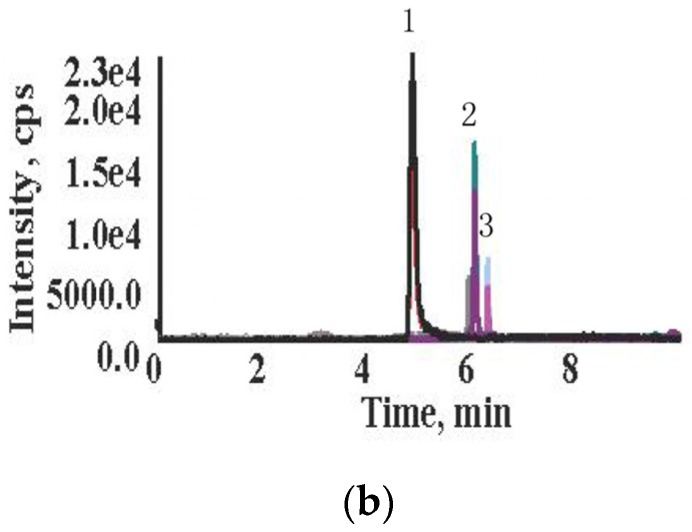
MRM chromatograms of (**a**) the four *Alternaria* toxins standards at concentrations of 250 μg/kg for TeA, 50 μg/kg for AOH, 50 μg/kg for TEN and 25 μg/kg for AME; (**b**) a naturally contaminated wheat sample with 112.9 μg/kg for TeA, 8.8 μg/kg for AOH, 3.3 μg/kg for TEN and negative for AME. 1 = TeA; 2 = AOH; 3 = TEN; 4 = AME.

The multiple reaction monitoring (MRM) chromatograms of the four *Alternaria* toxins standards and a naturally contaminated wheat sample are shown in Figure 1. The mean retention times (RTs) of the four toxins were 4.85 min for TeA, 6.12 min for TEN, 6.00 min for AOH and 6.37 min for AME, respectively. The RTs of these four toxins in wheat samples were set within ±2.5% difference with those of standards materials, in compliance with the requirements of European Union Decision 2002/657/EEC [17]. Excellent linearity of the standard curve was obtained for all four *Alternaria* toxins (coefficient *r^2^* > 0.99 for all curves). The limits of detection (LOD) and limits of quantification (LOQ) for each toxin in wheat samples were 0.6 and 1.9 μg/kg for TeA, 0.1 and 0.4 μg/kg for TEN, 1.3 and 4.2 μg/kg for AOH, and 0.04 and 0.1 μg/kg for AME, respectively (see Supplementary Table S1). The recoveries at five spiked concentrations ranged from 87.0% to 103.3% for all these four toxins in wheat samples, and the relative standard deviation (RSD) for all these four toxins were from 1.6% to 10.7%, which were both within the acceptable range recommended by the European Commission (Table 1) [18]. Regarding repeatability, the RSDr were 4.9% for TeA, 9.8% for TEN, 10.6% for AOH and 6.3% for AME, respectively. As for reproducibility, the RSD_R_ were 4.2% for TeA, 5.6% for TEN, 8.1% for AOH and 9.6% for AME, respectively. Based on the above results, it is concluded that the HPLC-MS/MS method for *Alternaria* toxins detection meets the requirements of our study.

### 2.2. Natural Occurrence of the Four Alternaria Toxins in Chinese Wheat

The contamination of the four *Alternaria* toxins in 370 freshly harvested wheat kernel samples are presented in Table 2. TeA is the predominant toxin in both frequency and concentration. It was found in all samples analyzed with an average level of 289.0 μg/kg (range: 6.0 μg/kg–3330.7 μg/kg, median = 150.0 μg/kg). Of all samples analyzed, 23 samples were contaminated with TeA at levels higher than 1000 μg/kg, 36 between 500 μg/kg and 1000 μg/kg, 184 between 100 μg/kg and 500 μg/kg, and 127 below 100 μg/kg. For TEN, 286 out of 370 (77%) samples were detectable, with levels ranging between 0.4 μg/kg and 258.6 μg/kg (mean = 43.8 μg/kg, median = 29.7 μg/kg). Among which, four samples were contaminated with TEN at a level higher than 200 μg/kg. One hundred and seventy three (47%) samples were positive for AOH, with a concentration range of 1.3 μg/kg–74.4 μg/kg (mean = 12.9 μg/kg, median = 7.9 μg/kg). There were three samples with AOH at a level higher than 50 μg/kg. AME was found in 55 (15%) samples at levels ranging from 0.3 μg/kg to 54.8 μg/kg (mean = 9.1 μg/kg, median = 4.2 μg/kg).

### 2.3. Geographical Distribution of the Four Alternaria Toxins in Anhui Province

**Figure 2:**
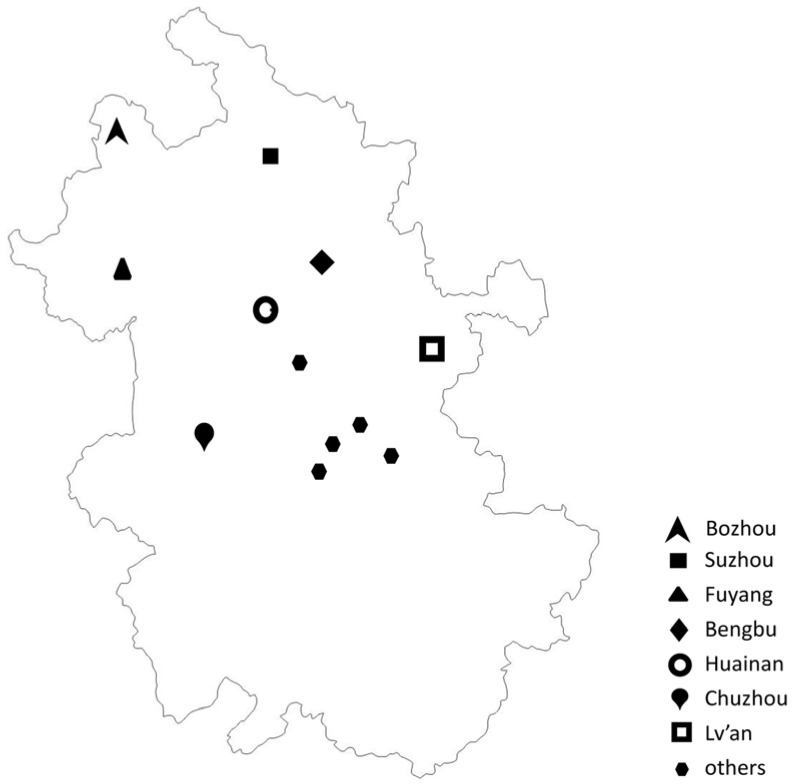
Geographical locations of eight sampling sites in Anhui province.

Wheat samples analyzed were collected from eight regions that covered almost all wheat-producing areas in Anhui province (Figure 2). The concentrations of the four *Alternaria* toxins in samples from different regions are shown in Table 3. It was demonstrated that the contamination of wheat kernels by the four *Alternaria* toxins varied geographically. The higher concentrations of the four toxins in terms of either average or median were found in samples from Fuyang, an area where, following a large scale flood, a major outbreak of human red mold intoxication caused by moldy wheat occurred in 1991, with 130, 141 people affected [14]. Fuyang is also adjacent to Henan, a province with high incidence of human esophageal cancer and where the contamination of *Alternaria* toxins in wheat-based products is relatively high [19]. Particularly, all (93/93) wheat samples from Fuyang were positive for TeA with a mean level of 582.6 μg/kg, which was significantly higher than those from the other regions (Mann-Whitney U Test, *p* < 0.05) except Huainan. TEN was detected in 99% (92/93) of wheat samples from Fuyang with an average level of 77.2 μg/kg, significantly higher than those from the other seven regions (*p* < 0.05). AOH was detected in 67% (62/93) of wheat samples from Fuyang, with a mean level of 18.3 μg/kg, significantly higher than those from the other regions (*p* < 0.05) except Chuzhou. Besides, 38% (35/93) of wheat samples from Fuyang were positive for AME with an average level of 8.5 μg/kg, significantly higher than those from the other regions (*p* < 0.05) except Huainan and Lv’an. The geographical distribution of the mean concentration of the four toxins (as well as most of the median) from high to low were in the following order : Fuyang > Huainan > Others > Bozhou > Lv’an > Chuzhou > Bengbu > Suzhou for TeA; Fuyang > Bozhou > Others > Bengbu > Chuzhou > Lv’an > Suzhou > Huainan for TEN; Huainan > Fuyang > Lv’an > Chuzhou > Others > Bozhou > Bengbu > Suzhou for AOH; Lv’an > Suzhou > Bengbu > Huainan > Fuyang > Chuzhou > Bozhou > Others for AME, respectively. Notably, the germination, growth and toxin production of *Alternaria* species would be influenced by environmental conditions [10]. Although these eight regions were all situated in Anhui province, the differences in temperature and precipitation among these areas during wheat earring, flowering, maturing and harvest season may lead to the significant differences in *Alternaria* toxin production.

### 2.4. Co-Occurrence of the Four Alternaria Toxins in Wheat Kernels

**Figure 3:**
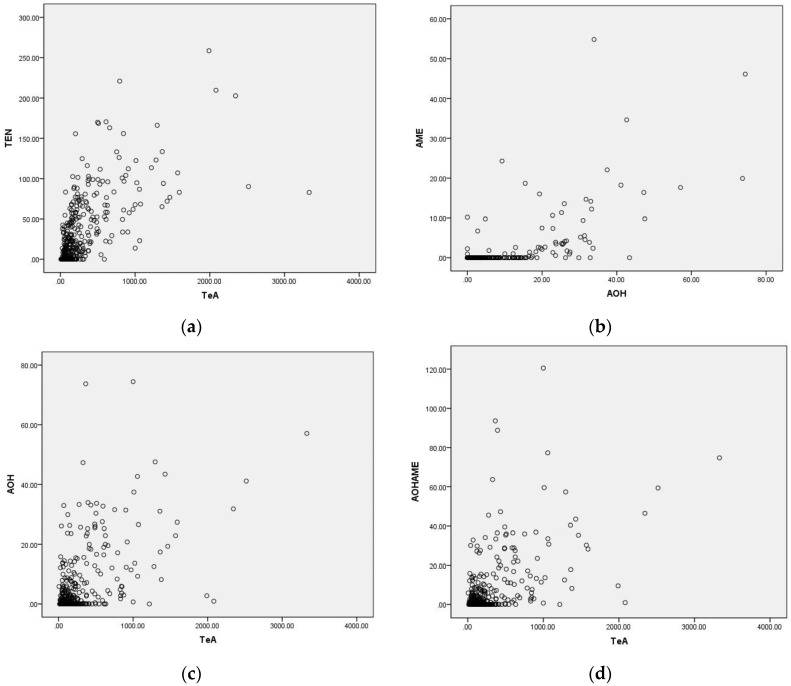
Correlation in concentrations of the four *Alternaria* toxins in wheat samples harvested in 2015 from Anhui province of China. (**a**) TeA vs. TEN, *r* = 0.675, *p* < 0.05, (**b**) AOH vs. AME, *r* = 0.558, *p* < 0.05, (**c**) TeA vs. AOH, *r* = 0.407, *p* < 0.05, (**d**) TeA vs. AOH + AME, *r* = 0.431, *p* < 0.05.

Co-occurrence of the four *Alternaria* toxins in wheat samples was frequent: 19% of samples (72/370) were contaminated by all four toxins, 30% (110/370) by three toxins, 46% (170/370) by two toxins and 5% (18/370) by only one toxin (TeA). Regarding the co-occurrence of the three toxins, 53 (48%, 53/110) samples were positive for TeA, TEN and AME, 20 (18%, 20/110) samples for TeA, TEN and AOH, 19 (17%, 19/110) samples for TeA, AOH and AME, and 18 (16%, 18/110) samples for TEN, AOH and AME, respectively. In terms of wheat co-contamination by the two *Alternaria* toxins, TeA and TEN was the most frequent combination detected in 60 (35%, 60/170) samples, followed by TeA and AME as well as TEN and AME, with the prevalence of 21% (35/170) for both combinations (Table 4). TeA and AOH occurred together in 14 (8%, 14/170) samples, whereas both AOH with TEN and AOH with AME in 13 (8%, 13/170) samples, respectively. Moreover, a significant linear correlation in concentrations was observed between TeA and TEN (*r* = 0.675, *p* < 0.05), AOH and AME (*r* = 0.558, *p* < 0.05), TeA and AOH (*r* = 0.407, *p* < 0.05), and also the total dibenzopyrone derivatives (AOH + AME) and TeA (*r* = 0.431, *p* < 0.05), respectively (Figure 3). These results are similar to those reported by Zhao et al. [19], of which a significant linear correlation in toxin concentration was found between AOH and AME (*r* = 0.877, *p* < 0.01), TeA and TEN (*r* = 0.747, *p* < 0.01), and the total dibenzopyrone derivatives (AOH + AME) and TeA (*r* = 0.860, *p* < 0.01).

## 3. Discussion

*Alternaria* species are widely distributed in various habitats such as the surface of buildings, cellulose, paper, and textiles as well as in the soil as normal components of its microflora [20]. They are also known to frequently occur in agricultural commodities. As *Alternaria* mycotoxins can contaminate agro-products across the food chain, and little information on their toxicity and occurrence is available, it has become an emerging concern for public health.

The contamination trends of the four *Alternaria* toxins in Chinese wheat analyzed in our study is in line with those described by the European Food Safety Authority (EFSA), of which TeA, TEN, AOH and AME were generally found in grain and grain-based products and TeA was the most predominant. When compared with the results from a 10-year study of a total of 1064 wheat samples between 2001 and 2010 conducted by German scientists [21], the prevalence of TeA (100%), AOH (47%) and AME (15%) found in the present study was much higher than that in German wheat (30%, 8% and 3%, respectively). However, the maximum levels of TeA, AOH and AME obtained in our study (3330.7 μg/kg, 74.4 μg/kg and 54.8 μg/kg, respectively) were lower than those reported in Germany (4224 μg/kg, 832 μg/kg and 905 μg/kg, respectively). To our knowledge, the highest concentrations of TeA, AOH and AME were found in Argentinean wheat at 8814 μg/kg for TeA, 1388 μg/kg for AOH and 7451 μg/kg for AME [22]. Although the contamination rates of TeA, AOH and AME in Chinese wheat were higher than those of Argentinean wheat samples (19% for TeA, 6% for AOH and 23% for AME), the average and maximum concentrations were much lower in our study. Thus, the geographical location plays an important role in toxin production.

In contrast to the average levels of *Alternaria* toxins in 22 Chinese weather-spoiled wheat samples (2419 μg/kg for TeA, 335 μg/kg for AOH and 443 μg/kg for AME) reported by Li et al. [23], the average levels of TeA, AOH and AME in this study were much lower. Moreover, our findings demonstrated that levels of TeA and TEN in wheat kernels were higher than those in wheat flour samples being collected also from Anhui province in 2014 as reported by Zhao et al. [19]. The mean and maximum levels of TeA in this current study were 2.4-fold and 6.4-fold, respectively, higher than those in wheat flour samples; for TEN it was 1.2-fold and 2.7-fod higher, respectively. Contrarily, AOH mean and maximum concentrations in wheat flour were 2.9-fold and 1.3-fold, respectively, higher than those of wheat kernels. Whereas, the AME concentration was very similar in both wheat kernel and wheat flour samples. The industrial procedures may be a factor in the different levels of the four *Alternaria* toxins among wheat kernel and wheat flour samples.

It is known that *Alternaria* toxin production is associated with the *Alternaria* species that invade, and their competition for energy with other fungal types [12]. *Alternaria* species are widespread in both humid and semi-arid regions. Furthermore, different species of *Alternaria* might be of different toxigenic profile. For example, the species of *Alternaria alternata* isolated from Argentinean wheat kernels could produce TeA, AOH and AME, but the species of *Alternaria infectoria* only produce TeA [24]. Interactions between *Alternaria* and *Fusarium* species and consequently their impact on toxin production has been reported by Muller et al. [25]. It was found that the increased population of *Alternaria* on ripening ears of wheat coincided with reduced population of *Fusarium* species [25,26]. Therefore, the increase of *Alternaria* toxins in cereals was often accompanied by the decline of *Fusarium* toxins.

The type of crop and variety of wheat as substrates for fungal growth, the agricultural practice and the climate condition might be responsible for the different levels of *Alternaria* toxins contamination in different geographical areas. Study shows that the contamination level of *Alternaria* toxins in freshly harvested wheat kernels was likely to be dependent on preceding year crop type and tillage [21]. Additionally, environmental conditions may influence the germination, growth and toxin production of *Alternaria* species. According to Magan et al. [27], the optimum temperature for *Alternaria* species invasion and toxin production was 25 °C with a water activity (*a*_w_) ranging between 0.88 and 0.99. The germination occurs within the temperature range of 5–35 °C, with the minimum *a*_w_ of 0.84–0.85 at 25 °C at pH 6.5. Therefore, a warm and humid climate during the period of wheat flowering, sprouting and heading stage (late April to June) in Anhui province is favorable to *Alternaria* fungal growth. These factors contribute to the different type of co-occurrence and correlation among *Alternaria* toxins observed between our research and the German study [21].

The study demonstrates a frequent presence of *Alternaria* toxins and the co-occurrence of four *Alternaria* toxins on wheat grain samples from Anhui province in China. The finding raises concern given the high wheat consumption in many regions in China. There is a need for more occurrence data to estimate the exposure to *Alternaria* toxins from cereal and cereal-based products in Chinese populations. Further, an integrated risk assessment of these four toxins’ contamination in Chinese wheat and the implications for public health needs to be carried out; strategies must be developed to reduce the risk from these contaminants, and specific regulations are expected to be established.

## 4. Materials and Methods

### 4.1. Chemicals and Reagents

Methanol and acetonitrile, both HPLC grade, were purchased from Fisher Scientific (Fair Lawn, NJ, USA). Ammonium bicarbonate and formic acid were of analytical grade (purity ≥ 95%) from Fluka (Steinheim, NRW, Germany). Pure water was obtained from a Millipore Milli-Q System (Millipore, Bedford, MA, USA). Standards of TeA (purity > 98%), TEN (purity > 98%), AOH (purity > 98%) and AME (purity > 98%) were supplied by Fermenteck Ltd. (Jerusalem, Israel). All experimental practice followed Environmental Health Safety Guidelines for the use of chemicals authorized by China National Center for Food Safety Risk Assessment. Samples extraction should be handled with care.

### 4.2. Samples Collection

A total of 370 wheat kernel samples were collected from local farmers who were randomly selected from main wheat production regions in Anhui province, China, namely Chuzhou, Fuyang, Huainan, Suzhou, Bozhou, Lv’an, Bengbu and a few “other” regions where a very small amount of samples were collected. All wheat samples were produced locally in 2015 and intended for human consumption. Fresh wheat kernels were sampled in the field during harvesting and threshing, one portion per household. Each portion was pooled from 4 subsamples (at least 600 grams each) that were collected from four areas of a field (north west, south west, north east and south east of the field). The sample were mixed thoroughly before a random quarter of the sample was selected, half of which was then finely ground to a powder of 20 meshes using a Blender 8010ES (Warning Commercial, Torrington, CT, USA). All ground samples were kept in the Ziploc plastic bags at 4 °C prior to analysis.

### 4.3. Toxin Analysis

All samples were analyzed for TeA, TEN, AOH and AME based on the methods reported previously by Zhao et al. [19] with modifications. Briefly, a finely ground sample (5 g) was mixed with 20 mL acetonitrile-water-methanol (45:45:10, *v*/*v*/*v*), adjusted pH between 3 and 4, and sonicated for 30 min before centrifugation. A 5 mL supernatant was loaded onto a HLB solid phase extraction (SPE) cartridge (Waters, Milford, MA, USA), eluted with 5 mL methanol followed by 5 mL acetonitrile. Both eluents were combined, dried and reconstituted in 1 mL methanol-water containing 2 mmol/L ammonium bicarbonate (10:90, *v*/*v*). Following centrifugation, 2 µL of the extract was analyzed for the four *Alternaria* toxins by HPLC-MS/MS method following the conditions described previously [28]. An *Alternaria* toxin-free wheat sample was used as a base for the matrix-matched calibration standards for quantification.

### 4.4. HPLC Conditions

HPLC-MS/MS system equipped with a Shimadzu 20A HPLC (Shimadzu, Kyoto, Japan), Triple Quard 3500 MS/MS system (AB Sciex, Foster City, CA, USA) was used to quantify the four *Alternaria* toxins simultaneously, and an Analyst Version 1.6.2 software (AB Sciex, Foster City, CA, USA) for data acquisition and analysis. Chromatographic separation was achieved using a C_18_ column (2.1 mm × 100 mm, 1.7 μm bead diameter, Waters, Milford, MA, USA). The temperature of both column and autosampler were set at 24 °C. A binary gradient with mobile phase A (2 mmol/L ammonium bicarbonate) and B (methanol) was programmed, and the flow rate was 0.3 mL/min.

### 4.5. MS/MS Conditions

The MS was operated in the negative electrospray ionization (ESI^−^) mode, set as MRM with an optimized dwell time of 50 ms. The curtain gas was set to 35 psi, the collision gas to 7 psi, Gas1 to 60 psi and Gas2 to 50 psi. The source desolvation temperature was 600 °C. The ion spray voltage was −4500 V in negative mode. Parent and fragment ions (quantifier and qualifier) for each analyte were determined based on the optimal signal-to-noise ratios in a spiked sample. The parent ions (*m/z*) for TeA, TEN, AOH and AME are 196.0, 413.2, 256.9 and 270.9, respectively. The most intense product ion was employed for quantification, and the less intense signals were used for confirmation of toxin identity. The quantitative/qualitative daughter ions are 138.8/111.8 for TeA, 140.9/271.0 for TEN, 212.9/214.9 for AOH and 255.8/227.8 for AME, respectively (see Supplementary Table S2).

### 4.6. Method Validation

To evaluate the method proficiency, *Alternaria* toxin-free wheat samples were spiked with the four *Alternaria* toxins at five concentrations ranging from 2 to 100 μg/kg for TeA, from 5 to 250 μg/kg for TEN, from 0.5 to 25 μg/kg for AOH, and from 0.5 to 10 μg/kg for AME, respectively. The spiked samples (*N* = 6 repeat each level) were extracted and analyzed for recovery calculation. In terms of the matrix-matched calibration, standards of the four *Alternaria* toxins were added to *Alternaria* toxin-free wheat extracts to reach the final concentrations between 2.5 and 5000 μg/kg for TeA, 0.5 and 1000 μg/kg for both TEN and AOH, 0.25 and 500 μg/kg for AME, respectively. The LOD and LOQ were determined based on the signal-to-noise ratios of 3:1 and 10:1, respectively. An *Alternaria* toxins-positive wheat sample was extracted and analyzed 6 times within 1 day for interlaboratory RSDr calculation. With regard to reproducibility, a naturally contaminated wheat sample was analyzed for *Alternaria* toxins over 5 successive days according to the procedure described above. The data obtained were used for RSD_R_ calculation.

### 4.7. Data Analysis

Significant differences in concentrations of mycotoxins were tested using Kruskal-Wallis H test or Mann-Whitney U test. Relationships of concentrations between any of the four mycotoxins were tested with the Spearman correlation. All statistical analyses were computed using the SPSS statistical package (version 20.0, IBM, Armonk, NY, USA).

## Figures and Tables

**Table 1:** The recovery (%) of the four *Alternaria* toxins in spiked wheat samples (No. of repeat = 6).

Mycotoxin	Spiked Level (μg/kg)	Recovery (%, $\bar{\chi }\pm S$)	RSD, %
TeA	2	95.3 ± 8.1	8.5
	10	95.8 ± 1.7	1.8
	20	98.9 ± 4.3	4.3
	40	96.3 ± 2.6	2.7
	100	99.3 ± 1.6	1.6
TEN	5	101.2 ± 4.1	4.1
	25	97.1 ± 5.4	5.6
	50	95.2 ± 3.4	3.6
	100	95.0 ± 4.7	4.9
	250	95.0 ± 2.6	2.7
AOH	0.5	103.3 ± 8.4	8.1
	2.5	95.3 ± 6.2	6.5
	5	97.6 ± 7.1	7.3
	10	98.1 ± 3.4	3.5
	25	96.2 ± 4.9	5.1
AME	0.5	91.7 ± 5.0	5.5
	1	87.0 ± 9.3	10.7
	2	100.3 ± 9.6	9.6
	5	94.0 ± 4.0	4.3
	10	98.1 ± 2.4	2.4

**Table 2:** Natural occurrence of the four *Alternaria* toxins in wheat kernel samples harvested in 2015 from Anhui province of China (*n* = 370).

Mycotoxin	Range (μg/kg)	Average (μg/kg)	Median (μg/kg)	Frequency, %
TeA	6.0–3330.7	289.0	150.0	100
TEN	0.4–258.6	43.8	29.7	77
AOH	1.3–74.4	12.9	7.9	47
AME	0.3–54.8	9.1	4.2	15

**Table 3:** Natural occurrence of the four *Alternaria* toxins in the 2015 wheat samples from different regions of Anhui province.

Region	Mycotoxin	Range (μg/kg)	Average (μg/kg)	Median (μg/kg)	Frequency, %
Chuzhou (*n* = 53)	TeA	6.0–3330.7	205.6	108.1	100 (53/53)
	TEN *	0.9–83.0	20.4	15.3	43 (23/53)
	AOH	1.3–57.1	12.1	7.8	87 (46/53)
	AME	2.1–17.6	6.2	3.9	9 (5/53)
Fuyang (*n* = 93)	TeA	53.7–2518.1	582.6	412.8	100 (93/93)
	TEN *	2.3–258.6	77.2	66.5	99 (92/93)
	AOH	1.3–74.4	18.3	16.0	67 (62/93)
	AME	0.3–46.1	8.5	4.2	38 (35/93)
Huainan (*n* = 13)	TeA	116.2–587.7	311.6	294.9	100 (13/13)
	TEN *	5.5–22.3	11.0	10.7	69 (9/13)
	AOH	4.3–47.3	26.8	26.6	46 (6/13)
	AME	1.4–16.4	8.8	10.6	38 (5/13)
Suzhou (*n* = 86)	TeA	13.2–832.3	125.0	86.5	100 (86/86)
	TEN *	0.4–76.7	14.2	8.7	58 (50/86)
	AOH	1.3–15.5	3.9	2.8	23 (20/86)
	AME	18.7	18.7	18.7	1 (1/86)
Bozhou (*n* = 35)	TeA	19.0–826.7	250.7	183.3	100 (35/35)
	TEN *	1.2–220.9	54.6	41.2	97 (34/35)
	AOH	1.6–30.3	6.5	3.2	46 (16/35)
	AME	0.9–9.8	4.2	3.1	11 (4/35)
Lv’an (*n* = 22)	TeA	21.1–1591.6	225.9	108.2	100 (22/22)
	TEN *	1.9–83.2	15.1	10.2	82 (18/22)
	AOH	1.3–33.9	12.5	9.6	50 (11/22)
	AME	0.9–54.8	20.7	13.4	18 (4/22)
Bengbu (*n* = 41)	TeA	21.2–616.2	131.1	93.4	100 (41/41)
	TEN *	0.5–101.5	21.6	14.1	80 (33/41)
	AOH	1.7–9.9	4.1	2.8	12 (5/41)
	AME	10.2	10.2	10.2	2 (1/41)
Others (*n* = 27)	TeA	47.3–1280.2	294.1	220.7	100 (27/27)
	TEN *	6.6–123.1	48.0	45.6	100 (27/27)
	AOH	2.3–12.8	7.3	7.5	26 (7/27)
	AME	0	0	0	0 (0/27)

* The concentration of TEN in samples from Fuyang was significantly higher than those from the other seven regions (*p* < 0.05).

**Table 4:** Co-occurrence of the four *Alternaria* toxins in wheat samples harvested in 2015 from Anhui province of China.

Contamination by	Toxin Combinations	Frequency, %
Two mycotoxins (46%, 170/370)	TeA-TEN	35 (60/170)
	TeA-AME	21 (35/170)
	TEN-AME	21 (35/170)
	TeA-AOH	8 (14/170)
	AOH-TEN	8 (13/170)
	AOH-AME	8 (13/170)
Three mycotoxins (30%, 110/370)	TeA-TEN-AME	48 (53/110)
	TeA-TEN-AOH	18 (20/110)
	TeA-AOH-AME	17 (19/110)
	TEN-AOH-AME	16 (18/110)
Four mycotoxins (19%, 72/370)	TeA-TEN-AOH-AME	19 (72/370)

## Supplementary Materials

The following are available online at www.mdpi.com/2072-6651/8/11/308/s1, Table S1: The parameters associated with the calibration curve and method sensitivity for the detection of the four *Alternaria* toxins, Table S2: The MRM parameters of MS/MS conditions.
