# Supplementary material for: Natural Occurrence of Alternaria Toxins in the 2015 Wheat from Anhui Province, China
Source: Toxins (Basel). 2016 Oct 26;8(11):308. doi: 10.3390/toxins8110308 (PMC5127105; doi:10.3390/toxins8110308)
Supplement: Supplementary file 1 [file toxins-08-00308-s001.pdf]

Supplementary Materials: Natural Occurrence of *Alternaria* Toxins in the 2015 Wheat from Anhui Province, China

Wenjing Xu, Xiaomin Han, Fengqin Li and Lishi Zhang

Table S1. The parameters associated with the calibration curve and method sensitivity for the detection of the four *Alternaria* toxins.

Mycotoxin [a]	Regressive Equation	Correlation Coefficient	LOD [b] (μg/kg)	LOQ [c] (μg/kg)
TeA	$Y = 1952.99\ X + 3227.47$	0.9999	0.6	1.9
TEN	$Y = 2598.29\ X + 51,067.2$	0.9972	0.1	0.4
AOH	$Y = 1859.83\ X + 4585.01$	0.9999	1.3	4.2
AME	$Y = 11388.87\ X + 138,459$	0.9953	0.04	0.1

[a] TeA = tenuazonic acid, TEN = tentoxin, AOH = alternariol, AME = alternariol methyl ether; [b] LOD = limit of detection; [c] LOQ = limit of quantification.

Table S2. The MRM parameters of MS/MS conditions.

Mycotoxin	Parent Ion	Daughter Ion	Dwell Time (ms)	DP [a] (v)	CE [b] (v)	CXP [c] (v)	EP [d] (v)
TeA	196.0	138.8	50	−87	−26	−10	−10
	196.0	111.8	50	−83	−32	−11	−10
TEN	413.2	140.9	50	−106	−26	−10	−10
	413.2	271.0	50	−106	−22	−11	−10
AOH	256.9	212.9	50	−114	−30	−7	−10
	256.9	214.9	50	−114	−34	−8	−10
AME	270.9	255.8	50	−106	−40	−10	−10
	270.9	227.8	50	−106	−38	−8	−10

[a] DP = declustering potential; [b] CE = collision energy; [c] CXP = collision cell exit potential; [d] EP = entrance potential.
